# Clinimetric Testing in Mexican Elders: Associations with Age, Gender, and Place of Residence

**DOI:** 10.3389/fmed.2014.00036

**Published:** 2014-10-17

**Authors:** Lorena Tavano-Colaizzi, Pedro Arroyo, Alvar Loria, Ana Bertha Pérez-Lizaur, Mario Ulises Pérez-Zepeda

**Affiliations:** ^1^Universidad Iberoamericana, Mexico City, Mexico; ^2^Instituto Nacional de Geriatría, Mexico City, Mexico; ^3^Instituto Nacional de Ciencias Médicas y Nutrición Salvador Zubirán, Mexico City, Mexico

**Keywords:** geriatric assessment, aged, clinimetrics, nursing homes, geriatrics

## Abstract

**Aim:** To evaluate the ability of five clinimetric instruments to discriminate between subjects >60 years of age living at home versus those living in a residency.

**Methods:** Trained nutritionists applied five instruments (cognition/depression/functionality/nutrition/appetite) to 285 subjects with majorities of women (64%), aged <80 years (61%), and home residents (54%).

**Results:** Multivariable regression models were generated for each instrument using age, gender, and residency as independent variables. Age was associated with worsening scores in the five instruments whereas residency showed association in three instruments, and gender in two. Score-age regressions by place of residency showed differences suggesting that Mundet residents had increasingly worse scores with increasing age than home dwellers for cognition, depression, and nutrition. Also, living at home prevented the worsening of depression with increasing age. In contrast, functionality and appetite deteriorated at a similar rate for home and Mundet residents suggesting an inability of these two instruments to discriminate between settings. Score-age regressions by gender suggested that males have less cognitive problems at 60 and 80 years of age but not at 100 years, and better appetite than women at all ages.

**Conclusion:** Increasing age proved to be associated to worsening scores in the five instruments but only three were able to detect differences according to setting. An interesting observation was that living at home appeared to prevent the depression increase with increasing age seen in Mundet residents.

## Introduction

Functional decline of older adults is a condition that affects different health domains, including physical, cognitive, psychological, and nutritional aspects (1). The rate of decline is a function of age, modulated by multiple factors, including gender and environment (2). The health status of elderly people is usually screened with tools, which differ according to domain and purpose: there are screening, predictive, and diagnostic tests dealing with cognitive decline, depression, functional dependency, and nutritional assessment (3). These instruments are widely used and have been standardized in different populations and moments, both in clinical and research settings.

There is scarce literature in regard to clinimetric testing in general populations in Mexico, especially old adults. We have previously reported the results of a single clinimetric test, the micro-nutrimental assessment (MNA) test, in community-dwelling and institutionalized adults with ages above 60 years but did not take into account differences between groups in gender and age (4). The present study compares home dwellers versus institutionalized residents in five clinimetric tests (MNA plus cognitive deterioration, depression, functionality, and appetite) taking into account age and gender of the participants. Our aim was to explore, which of these clinimetric tests were able to distinguish elders living at home from elders living in an institutional residency.

## Materials and Methods

A total of 285 subjects, 183 women/102 men, aged 60–102 years (mean = 76.0) participated. One hundred seventy three subjects were living at the Arturo Mundet Residency for the Aged located in the Administrative Zone Álvaro Obregón in Mexico City, and the remainder were living at home and attended one of eight day-care centers (1. Casa del Adulto Mayor; 2. Centro de Desarrollo Social Torres de Potreros; 3. Centro de Atención Integral Zenón Delgado; 4. Casa del Adulto Mayor Gloria; 5. Casa del Adulto Mayor Amor a la Alegría; 6. Casa Jenner; 7. Casa Luz de Primavera; 8. Casa La Asunción). They were located in the same administrative zone as the Mundet Residency. The interviews were performed by nutritionists who had completed a 20-h theoretical and practical course on the questionnaire. The questionnaire included age and sex of the participants plus the questions of five clinimetric tests designed to evaluate cognitive deterioration, depression, functionality, nutrition, and appetite. The instruments were applied in this same order during a single session.

The cognitive deterioration test of Pfeiffer (5) counts the number of wrong answers to 10 questions: thereby the score ranges between 0 (No deterioration) to 10 (Severe deterioration). The geriatric depression scale of Sheikh and Yesavage (6) has 15 items in which the yes/no answers are scored as 1 or 0, and the score ranges from 0 (No depression) to 15 (established depression). The scores of these two instruments are directly proportional to the respondent’s derangement, thus, high scores indicate poor cognitive status and severe depression, whereas for the other instruments, a high score indicates good functionality, nutrition, and appetite. The functionality test of Mahoney and Barthel (7) relates to self-care activities of daily life and the ability to move around and use stairs. It has 10 questions with a variable number of answering scores: 2 questions had two scores (0 and 5), 6 questions had three scores (0, 5, 10), and 2 with four scores (0, 5, 10, 15), and the sum of the question scores ranges from 0 (totally dependent) to 100 (totally independent). Nutritional status was assessed using the Mini Nutritional Assessment Scale (MNA) of Guigoz et al. (8), which has 18 items, each scored from zero to a variable maximum score (6 items with a maximum of 1 point; 9 items with a maximum of 2 points; and 3 with maximum of 3), so that the overall maximum score was 33 (well nourished). The Appetite test of Wilson et al. (9) has 8 items, each with a 1–5 Likert scale so the scores range between 8 (very poor appetite) and 40 (very good appetite): Wilson et al. (9) dichotomize the score so that a score of 28 or more is indicative of good appetite, and a score below 28 indicates that the individual is at risk of suffering body weight loss of 5% or more in the next 6 months. The authors of the other tests have proposed three to five categories for their scores but we used the raw scores in all our analysis.

The research was approved by the Ethics and Research Committee of the Universidad Iberoamericana of Mexico City and consent was signed by the participants.

### Statistical analysis

Mean and SD were used to summarize the raw scores of the five instruments. Group differences were evaluated with the chi square test for sex and place of residency, and with the *t* test for age and scores. The regression analysis was done using the General Linear Modeling method of SPSS version 21. The slope differences of the regression equations were analyzed with a procedure of the UCLA Statistical Consulting Group (10). A two-tailed *P* ≤ 0.05 was considered significant.

## Results

The 285 participants had majorities of women (183/285 = 64%), below 80 years of age (173/285 = 61%) and living at home (155/285 = 54%).

### Univariate analysis

The mean scores of the five instruments (cognitive deterioration/depression/functional independency/nutritional assessment/appetite) are given in Table 1 together with the group differences associated with age, sex, and residency (yes/no living in the Arturo Mundet Residency for the Aged) (Table 1).

**Table 1:** **Group differences of raw scores (mean ± SD) for the five clinimetric tests in the 285 subjects**.

Clinimetric test	Age in years		*P*-value
	60–79 (*N* = 173)	80–102 (*N* = 112)	
Cognitive	1.2 ± 1.5	3.0 ± 2.6	0.005
Depression	2.7 ± 2.5	3.6 ± 2.9	0.009
Functional	96.0 ± 9.4	89.2 ± 17.3	<0.001
Nutritional	24.9 ± 3.5	22.4 ± 4.3	<0.001
Appetite	29.7 ± 3.3	28.7 ± 3.6	0.02

	**Living at Mundet residency**		
	No (*N* = 155)	Yes (*N* = 130)	

Cognitive	1.1 ± 1.3	2.9 ± 2.6	<0.001
Depression	2.6 ± 2.4	3.6 ± 3.0	0.002
Functional	97.7 ± 4.5	88.2 ± 18.0	<0.001
Nutritional	24.8 ± 3.6	22.9 ± 4.3	<0.001
Appetite	29.6 ± 3.6	28.9 ± 3.2	NSD 0.09

	**Gender**		
	Females (*N* = 183)	Males (*N* = 102)	

Cognitive	2.0 ± 2.2	1.8 ± 2.2	NSD 0.30
Depression	3.0 ± 2.8	3.1 ± 2.7	NSD 0.94
Functional	93.2 ± 13.1	93.6 ± 14.2	NSD 0.81
Nutritional	23.8 ± 3.9	24.1 ± 4.3	NSD 0.55
Appetite	29.0 ± 3.6	29.8 ± 3.2	NSD 0.09

*Significance of group differences*.

*NSD, non-significant difference*.

*Groups made by age, place of residency, and gender*.

Being older than 80 years of age was significantly associated with poorer performance in all five instruments, and with four instruments according to place of residency, i.e., there were higher scores for the first two instruments inversely related with performance (cognitive deterioration and depression) and lower scores for the three instruments directly related with performance. In contrast, there were no score differences by gender in the univariate analysis.

### Multivariable analysis

A backward multivariable analysis was carried out with the raw scores of each instrument as the dependent variable, and age, sex, and residency as the independent variables (Table 2). Table 2 shows the regression equations for each test. Age was the only variable that entered all five models following the pattern seen in the univariate analysis of worsening scores with increasing age. On the other hand, living at the Mundet Residency was not significantly associated with the Nutritional and Appetite scores in the multivariable analysis despite their significance in the univariate analysis and, in contrast, gender now appeared significantly associated with cognition and appetite (females performing worse than males in both instances).

**Table 2:** **Multivariable regression equations of the test scores using age, sex, and living at the Arturo Mundet Residency as independent variables**.

Clinimetric test	Regression equation	Effect on scores due to		
		Increasing age	Mundet residence	Being female
Cognitive	−4.9 + 0.086 age + 1.06 Mundet − 0.45 male	Worse	Worse	Worse
Depression	0.09 + 0.035 age + 0.70 Mundet	Worse	Worse	–
Functional	108.2 − 0.147 age − 8.16 Mundet	Worse	Worse	–
Nutritional	35.3 − 0.150 age	Worse	–	–
Appetite	33.5 − 0.059 age + 0.75 male	Worse	–	Worse

*Age was entered in the models as a continuous variable*.

To illustrate the use of the regression models of Table 2, we shall use the simplest equation – that of the Nutritional test – so that the multiplication of the age coefficient (0.150) by 60, 80, and 100 years of age gives values of 9, 12, and 15, respectively. These were subtracted to the intercept value of 35.3 (subtracted because the age coefficient is negative) to render scores of 26.3, 23.3, and 20.3 expected for the nutrition instrument at those ages.

### Regression equations according to residency

The equations of scores versus age in subjects living at home versus those in the Mundet Residency are shown in Table 3. The slope of the equation in the Mundet residents was significantly higher for three of the five tests. In addition, the Depression slope (0.001) in home dwellers was the only one that was not significantly different from zero. This means that the increasing depression score with increasing age seen in the Mundet residents, was absent in those living at home.

**Table 3:** **Regression equations of scores versus age in two groups made by place of residency (home versus Mundet residency)**.

Clinimetric test	Home (*N* = 155)	Mundet (*N* = 130)	*P* values
	Slope
Cognitive	−2.4 + 0.049 age	−7.9 + 0.134 age	0.001
Depression	2.5 + 0.001 age^a^	2.6 + 0.077 age	0.04
Functional	102.8 − 0.070 age	107.9 − 0.243 age	NSD 0.32
Nutritional	31.2 − 0.089 age	38.2 − 0.189 age	0.05
Apetite	33.6 − 0.058 age	33.1 − 0.052 age	NSD 0.93

*Significance of group difference in slope*.

*NSD, non-significant difference*.

*^a^Only equation with slope not significantly different from zero*.

**Figure 1:**
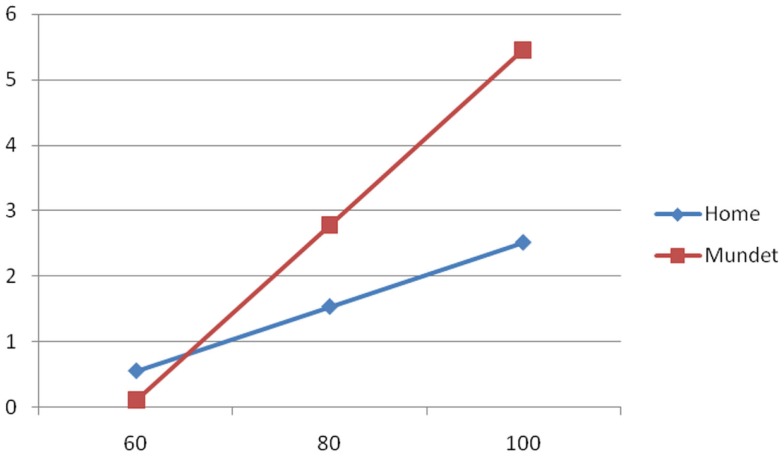
**Cognitive score (*Y* axis) versus age in years (*X* axis) in home and Mundet residents**.

**Figure 2:**
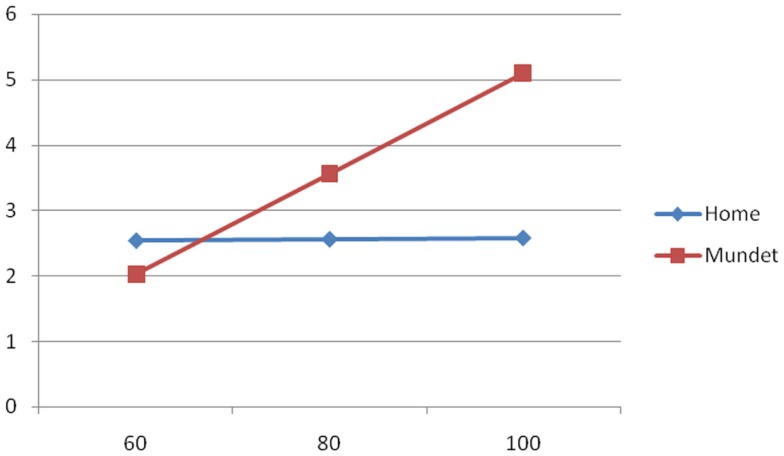
**Depression score (*Y* axis) versus age in years (*X* axis) in home and Mundet residents**.

**Figure 3:**
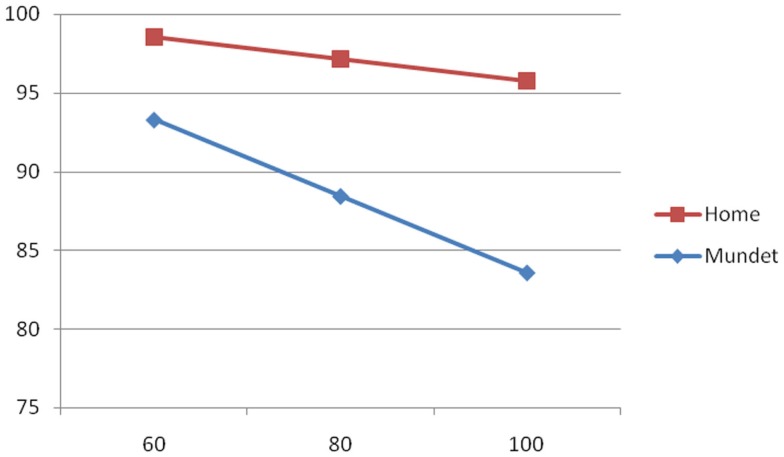
**Functionality score (*Y* axis) versus age in years (*X* axis) in home and Mundet residents**.

To illustrate the group differences of the equations of Table 3, Figures 1–3 show the behavior of the scores at 60, 80, and 100 years of age for the cognitive, depression, and functionality tests, respectively. The three figures show that scores in the Mundet residents were worse than in home dwellers. In addition, Figure 2 agrees with the near-zero slope of the regression in subjects living at home and confirms that the increasing depression that accompanied age in the institutionalized group was not seen in those living at home. Figure 3 shows that the Mundet residents had a mean functional score below 95 at age 60 years whereas home dwellers were never below 95 not even at age 100 years. This group difference goes along the popular saying in Mexico that if they can afford it, elderly people are institutionalized as soon as they become functionally dependent.

The regressions of score by age in males and females (data not shown) showed that males had less cognitive problems at 60 and 80 years of age (but not at 100), and better appetite than women at all ages.

## Discussion

In our analysis we decided to use the raw scores and not the categorized scores proposed by the researchers that developed the instruments. We adopted this approach as we have little information on how they behave in Mexican elders that are culturally different from the populations in which the score categorizations were established. In addition, the larger distribution range of raw scores versus categorized scores enables them to be considered as continuous variables – in a manner similar to what is habitually done with age – making them convenient for regression modeling. Finally, the presence of outliers that could unduly affect our regressions was discarded by the examination of scatterplots of age (*X*) versus score (*Y*) that showed diffuse monovalent distributions without outliers in the five instruments. Straight lines adjusted to a diffuse distribution are sufficient to detect trends in regression analysis (11) such as the trend observed in this study, i.e., worsening scores with increasing age in subjects with more than 60 years of age.

Our multivariable analysis confirmed that the five instruments were significantly associated with age, considered the most important factor influencing the rate of decline in aged populations (12). The fact that we were able to establish these age associations in the five tests supports our decision to use raw scores in our analysis, i.e., the age association was lost using the binary categorization of scores (<28 versus ≥28) proposed by Wilson et al. (9) for their Appetite test. The data of Table 2 confirmed the age effect on the performance of all five instruments with worsening scores as age increased, despite the fact that our population was not a hospitalized population and could be considered to being in good health. This may imply the need to consider different cut-off points according to age, when interpreting the results of these five clinimetric tests in subjects having more than 60 years of age.

Place of residency on the other hand, showed association only with cognition, depression, and nutrition. The differences between institutionalized residents versus home dwellers in these instruments was a finding to be expected (2, 13) especially as our institutionalized residents were compared with home dwellers still able to attend a day-care center. The latter were presumably at a lower risk of presenting cognitive or psychological problems than institutionalized residents, and these differences were discriminated by three of the five instruments. In contrast, the functional and appetite instruments were unable to detect a significant difference between home and institutionalized elders, a finding that may have been due to other causes, such as masking by age and gender.

Regarding gender, males scored better than females in cognition and appetite. In our view, the poorer cognition in females can be partially explained by lack of schooling especially for women born before 1950 when primary school was not mandatory in Mexico, and most girls grew up unschooled. The lower appetite in females, on the other hand, was somewhat unexpected as in Mexico, women usually are the decision makers of household menus and presumably cook their food preferences and thus, one would expect a better appetite in women. The only explanation that comes to our mind is that women are rarely stimulated to eat food – especially food cooked by herself – whereas men are offered and stimulated to eat food cooked by a wife at home or by the mostly female kitchen personnel at the Mundet institution.

## Conflict of Interest Statement

The authors declare that the research was conducted in the absence of any commercial or financial relationships that could be construed as a potential conflict of interest.
